# Influence of Different Drying Techniques on the Drying Kinetics, Total Bioactive Compounds, Anthocyanin Profile, Color, and Microstructural Properties of Blueberry Fruit

**DOI:** 10.1021/acsomega.3c05749

**Published:** 2023-10-26

**Authors:** Alican Akcicek, Esra Avci, Zeynep Hazal Tekin-Cakmak, Muhammed Zahid Kasapoglu, Osman Sagdic, Salih Karasu

**Affiliations:** † Faculty of Tourism Department of Gastronomy and Culinary Arts, Kocaeli University, Kartepe, 41080 Kocaeli, Turkey; ‡ Department of Food Engineering, Faculty of Chemical and Metallurgical Engineering, Yildiz Technical University, Davutpasa Campüs, 34210 Istanbul, Turkey; § Bypro Functional Food and Biotechnology, Esenler, 34210 Istanbul, Turkey; ∥ Istanbul Teknokent, Cerrahpasa Avcılar Campus, Istanbul University, 34452 Istanbul, Turkey

## Abstract

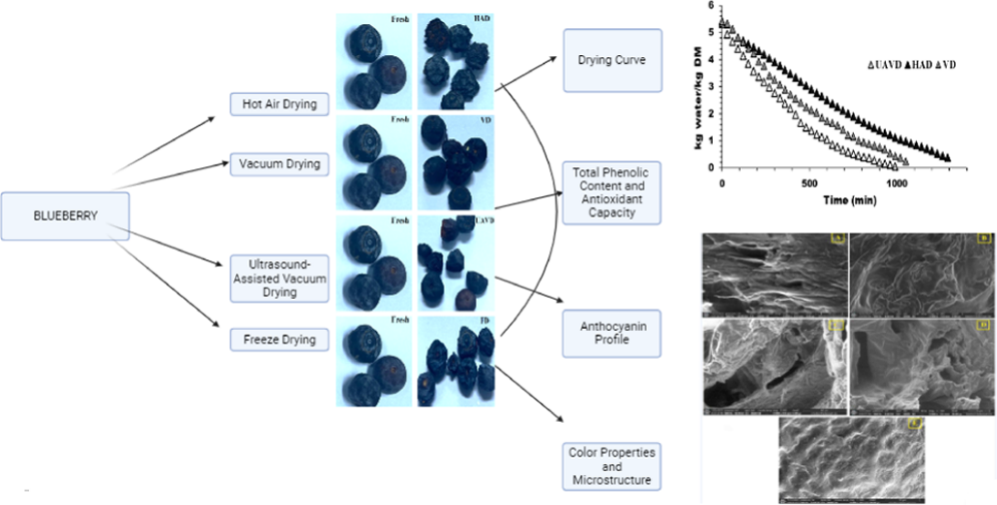

In this study, four different drying techniques, namely, hot air drying (HAD), vacuum drying (VD), ultrasound-assisted vacuum drying (UAVD), and freeze-drying (FD), were applied to blueberries. The drying times of blueberries were 1290, 1050, and 990 min for HAD, VD, and UAVD, respectively, meaning that ultrasound application significantly reduced the drying time. All dried samples except those with FD showed lower total phenolic content and antioxidant capacity than fresh samples. Samples dried with FD had a higher content of bioactive compounds than those dried with other techniques followed by UAVD. The malvidin-3-*O*-galactoside was the most abundant anthocyanin in the blueberries and was significantly reduced after drying with HAD, VD, and UAVD. Scanning electron microscopy (SEM) analysis of the blueberries dried with FD and UAVD exhibited less shrinkage and cell disruption and more structure. The color parameters L*, a*, and b* values of the samples were significantly affected by the drying technique (*p* < 0.05). According to the findings of this study, ultrasound-assisted drying technology could be employed to shorten the drying time and improve bioactive retention in blueberry fruits.

## 1 Introduction

*Vaccinium myrtillus* known as blueberry is a plant native to North America and commonly consumed due to its high nutritional value and flavor.^1,2^ These berries have strong free radical scavenging characteristics owing to bioactive compounds such as anthocyanin and polyphenols.^3^ Blueberries have anti-inflammatory, antibacterial, anticarcinogenic, and antioxidant actions, which reduce oxidative stress and risk of diseases and prevent cardiovascular disease.^4^ However, the shelf life and the consumption of blueberries are restricted due to the seasonal availability.^5^ Furthermore, even under low-temperature storage environments, fresh blueberries are extremely vulnerable to microbial contamination.^6^ These challenges of the blueberries need to be overcome by using the processing for the availability out of the season.^3^

Drying is a food processing technique that expands the shelf life by reducing water activity and the mass of the product.^5^ The drying reduces spoilage and contamination and thus assures quality and stability.^7^ In addition, drying enables easy packaging, handling, and transportation.^8^ Hot air drying is a widely used drying method since it is inexpensive and simple to use. The drawbacks of hot air drying, however, are the prolonged drying time, exposure to oxidation, and production of off odors.^9^ When compared to alternative drying techniques, freeze-drying ensures the production of fruit and vegetable products of the highest quality.^10^ Despite its long drying time and high costs, freeze-drying remains widely used in producing high-value food items due to its exceptional retention of food quality compared to other drying techniques.^11^ However, new alternative drying techniques should be researched to overcome these challenges such as long drying time, high cost, and slow process.

Another common method for drying berries is vacuum drying (VD), which has benefits such faster drying rate, lower operating temperatures, and capacity to work in a low-oxygen atmosphere.^12,13^ Drying technology has advanced from a single drying technique (such as hot air drying or vacuum drying) to a combination of drying processes. These methods offer benefits, including great effectiveness, affordability, adaptability, and environmental friendliness.

The ultrasound-assisted vacuum drying (UAVD) technology is generally used due to shortened drying time and developed drying efficiency by increasing the dehydration rate without heating up under vacuum.^14^ Ultrasound has the capability to improve internal moisture transfer and induce cavitation, and ultrasonic waves generate tiny channels, facilitating water removal. Moreover, the ultrasound also creates cavities that help eliminate tightly bound water within the material, without causing significant heating.^15,16^ This approach helps to preserve heat-sensitive food ingredients. Red peppers,^17^ salmon and trout fillets,^18^ nectarines,^19^ carrot slices,^20^ raspberry fruit,^21^ Schisandra chinensis extract powder,^22^ papaya,^23^ and flos sophorae immaturus^16^ are the food products that are processed by UAVD.

In this research, blueberry fruit was subjected to different drying methods, which are hot air drying (HAD), ultrasound-assisted vacuum drying (UAVD), and freeze-drying (FD). The study aimed to examine the influence of these drying techniques on drying kinetics, total phenolic content, antioxidant activity, microstructural properties, anthocyanin content, and color properties.

## 2 Material and Methods

### 2.1 Material

Blueberry was bought from a local grocery store. The initial moisture content was determined as 83.95 ± 1.15% using a vacuum oven set at 70 °C for 6 h.^24^ Approximately 20 fresh blueberries (30 g) were used for each treatment.

### 2.2 Drying Experiments

Blueberries were subjected to drying using four different methods: hot air drying (HAD), ultrasound-assisted drying (UAVD), vacuum drying (VD), and freeze-drying (FD). All methods except for FD involved drying at a temperature of 50 °C. The hot air drying (HAD) process was carried out at a constant air speed of 1.3 m/s, and the air velocity was measured using a Testo 440 vane probe anemometer (Lutron, AM-4201, Taiwan). The air flowed horizontally over the surface of the blueberries during this process. Blueberries were vacuum-dried by using a vacuum drier (DaihanWOV-30, Gangwon-do, Republic of Korea). In the VD and USVD methods, a vacuum pump (EVP 2XZ-2C, Zhejiang, China) with an ultimate pressure of 60 mbar and a pump speed of 2 L/s was used to control the vacuum level. UAVD was conducted following the method described by ref (25). For the ultrasound-assisted vacuum drying (UAVD) method, the system consisted of a combination of a South Korean Daihan WUC-D10H ultrasonic water bath (100% amplitude, 2 power density, 10 L volume) and a German KNF N838.3KT.45.18 vacuum pump (15 mbar pressure, 22 L/min speed). The blueberry samples were placed in a flask connected to a vacuum pump and sonicated using an ultrasonic water bath at a frequency of 40 kHz. In the case of freeze-drying (FD), the blueberries were first frozen at −80 °C for 24 h and then dried using a freeze-drying program with a Martin Christ β 1-8 LSCplus freeze-drying machine. The blueberry fruit weight loss was monitored every 30 min for HAD, VD, and USVD, and the drying procedure continued until the fruits’ final moisture content was 0.2 kg water/kg dry basis (d.b).

#### 2.2.1 Modeling of the Dehydration Characteristic

Nine thin-layer drying models were used for modeling the data, including Lewis, Henderson and Pabis, logarithmic, Wang and Singh, two-term exponential, parabolic, and Weibull (Table 1). As the value of *M*_e_ is very small in comparison to *M* or *M*_0_, the moisture ratio (MR), where *M* is the moisture content at time *t*, *M*_0_ is the initial moisture content, and *M*_e_ is the equilibrium moisture content, was simplified in accordance with these models to be *M*/*M*_0_ rather than (*M* – *M*_e_)/(*M*_0_ – *M*_e_).

**Table 1:** Mathematical Models Used to Explain Drying Kinetics

model name	model	references
Lewis	*y* = exp(−*k* × *t*)	(26)
Henderson and Pabis	*y* = *a* × exp(−*k* × *t*)	(27)
logarithmic	*y* = *a* × exp(−*k* × *t*) + *c*	(28)
two term	*y* = *a* × exp(−*c* × *t*) + *b* × exp(−*d* × *t*)	(29)
Wang and Singh	*y* = 1 + *a* × *t* + *b* × *t*^2^	(30)
two-term exp	*y* = *a* × exp(−*k* × *t*) + (1 – *a*) × exp(−*k* × *a* × *t*)	(31)
parabolic	*y* = *a* + *b* × *t* + *c* × *t*^2^	(32)
Weibull	*y* = exp(−(*t^a^*/*b^a^*))	(33)

The drying rate (DR) was calculated using eq 1
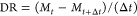
1where *M_t_* + Δ*t* is the moisture content at *t* + Δ*t* (kg water/kg dry matter) and *t* and Δ*t* are time (min). A nonlinear regression analysis was used to determine the model parameters and *R*^2^ values using Statistica software (StatSoft, Tulsa). *R*^2^ and root-mean-square error (RMSE) metrics were used to assess the model acceptance. Higher *R*^2^ and lower RMSE values suggest that the constructed model is suited for purpose. RMSE values were obtained using eq 2:^34^
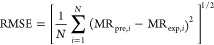
2In eq 2, MR_prei_ is the predicted moisture ratio, MR_expi_ is the experimental moisture ratio, *N* is the number of observations, and *n* is the number of constants.

### 2.3 Extraction

Extraction of bioactive compounds was carried out using a methanol/water (1:1) mixture. For the extraction process, 1 g of sample was transferred to a test tube and 10 mL of methanol–water mixture was added. The mixture was homogenized with a Daihan HG-15D model Ultraturrax device at 10 000 rpm for 2 min. The mixture was then shaken at 5000 rpm for 1 h at room temperature and separated by centrifugation at 5000 rpm for 10 min. The resulting supernatant was filtered using a 0.45 μm syringe filter, and the resulting extracts were stored at −20 °C for further analysis.

### 2.4 Determination of the Total Phenolic Content

The total phenolic content of raspberries was assessed using a modified method as described by Singleton and Rossi.^63^ For this analysis, 0.5 mL of the extract, 2.5 mL of a 10-fold diluted Folin Ciocalteu’s phenol reagent, and 2 mL of Na_2_CO_3_ (7.5%) were combined. The mixture was left to incubate for 30 min at room temperature in a dark environment. Subsequently, the absorbance was measured at 760 nm by using a UV/vis spectrophotometer (Shimadzu UV-1800, Kyoto, Japan). The results were expressed as milligrams of gallic acid equivalent (GAE) per gram of dry matter (DM) (mg of GAE/g DM).

### 2.5 Determination of Antioxidant Capacity (DPPH and ABTS)

DPPH analysis was performed by mixing each sample with 0.1 mL of DPPH (1,1-diphenyl-2-picrylhydrazil) solution. After an incubation period of 60 min, the absorbance of the samples was measured at 517 nm using a spectrophotometer (Shimadzu UV-1800, Japan). The results are expressed in amounts equivalent to Trolox. To determine the ABTS radical scavenging activity, a 7 mM ABTS solution containing 2.45 mM potassium persulfate was initially prepared. The formation of ABTS^+^ radicals was achieved by allowing the solution to stand at room temperature for 12–16 h. Before the analysis began, 1 mL of the ABTS+ radical solution was extracted, and its absorbance value was adjusted to 0.700 ± 0.02 at 734 nm through dilution. Subsequently, 2 mL of the diluted ABTS+ radical solution was transferred into a microcuvette, followed by the addition of 100 μL of the hydrolyzate solution. The microcuvette was then kept in darkness for 6 min before measurements were taken using a spectrophotometer (Shimadzu UV-1800 UV/vis, Tokyo, Japan). The results are presented as amounts equivalent to Trolox.

### 2.6 Analysis of Individual Anthocyanin Profile

Anthocyanin compounds were analyzed according to the HPLC procedure described by ref (35) with a few modifications. A Shimadzu LC-20A HPLC system (Kyoto, Japan) consisting of a binary pump (LC-20AT), a UV–vis photodiode-array detector (SPD-M20A), and a column oven (CTO-10AS) was used for the analyses of anthocyanins. Initially, sample extracts were centrifuged, diluted, and then filtered using a 0.45 μm Millipore filter before HPLC analyses. HPLC analyses were performed by using a 5 μm Inertsil ODS-3 C18 column (250 × 4.6 mm^2^; GL Sciences). The injection volume of the samples in HPLC-DAD was 20 μL. The separation was carried out with a mobile phase consisting of solvent A (water/formic acid, 93/7) and solvent B (acetonitrile/water/methanol, 90/5/5) under gradient conditions at 1.2 mL/min. The column was maintained at 25 °C. The chromatograms were recorded at 520 nm for anthocyanin and 320 nm for other phenolic compounds. All mobile phases were filtered by using a 0.45 μm Millipore filter and degassed in the ultrasonic bath. The results for anthocyanins were expressed as milligrams of cyanidin-3-*O*-glucoside equivalent (mg C3GE)/L of the sample.

### 2.7 Color Measurement

The color of the blueberry samples was evaluated by using a colorimeter (CR-400 Konica, Minolta, Tokyo, Japan). The color values of the samples were expressed by using the L*, a*, and b* parameters, which represent brightness/darkness, redness/greenness, and yellowness/blueness, respectively. After calibrating at a standard illuminant (D65, 10° observer angle), the L*, a*, and b* characteristics of the samples were measured. The following CIEDE 2000 equation was used to express and assess the samples’ overall color change:
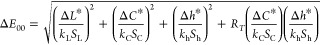
3ΔL*, ΔC*, and Δh* are the CIELAB metric lightness, chroma, and hue differences, respectively; *k*_L_, *k*_C_, and *k*_h_ values are the parametric factors; *S*_L_, *S*_C_, and *S*_h_ are the weighting functions for the lightness, chroma, and hue components, respectively.

### 2.8 Environmental Scanning Electron Microscopy (ESEM)

Environmental scanning electron microscopy (ESEM) was used to capture images of the cross-sectional microstructure inside both dried and fresh blueberries. First, the blueberry samples were cut in half by using a knife. Subsequently, an environmental scanning electron microscope (ESEM) operating at 3 kV (Thermo Scientific Quattro S) was employed to acquire the images.

### 2.9 Statistical Evaluation

The data was subjected to statistical analysis using the JMP 9 software (SAS, NC). The relevant variables were computed for their arithmetic mean and standard deviations. To identify any significant differences between the variables, a one-way analysis of variance (ANOVA) along with the Tukey test was employed. Based on the analysis results, a statistically significant difference among the variables was observed at the significance level of *p* < 0.05 (typically, a significance level of 0.05 is considered).

## 3 Result and Discussion

### 3.1 Effect of Drying Methods on Drying Time

**Figure 1:**
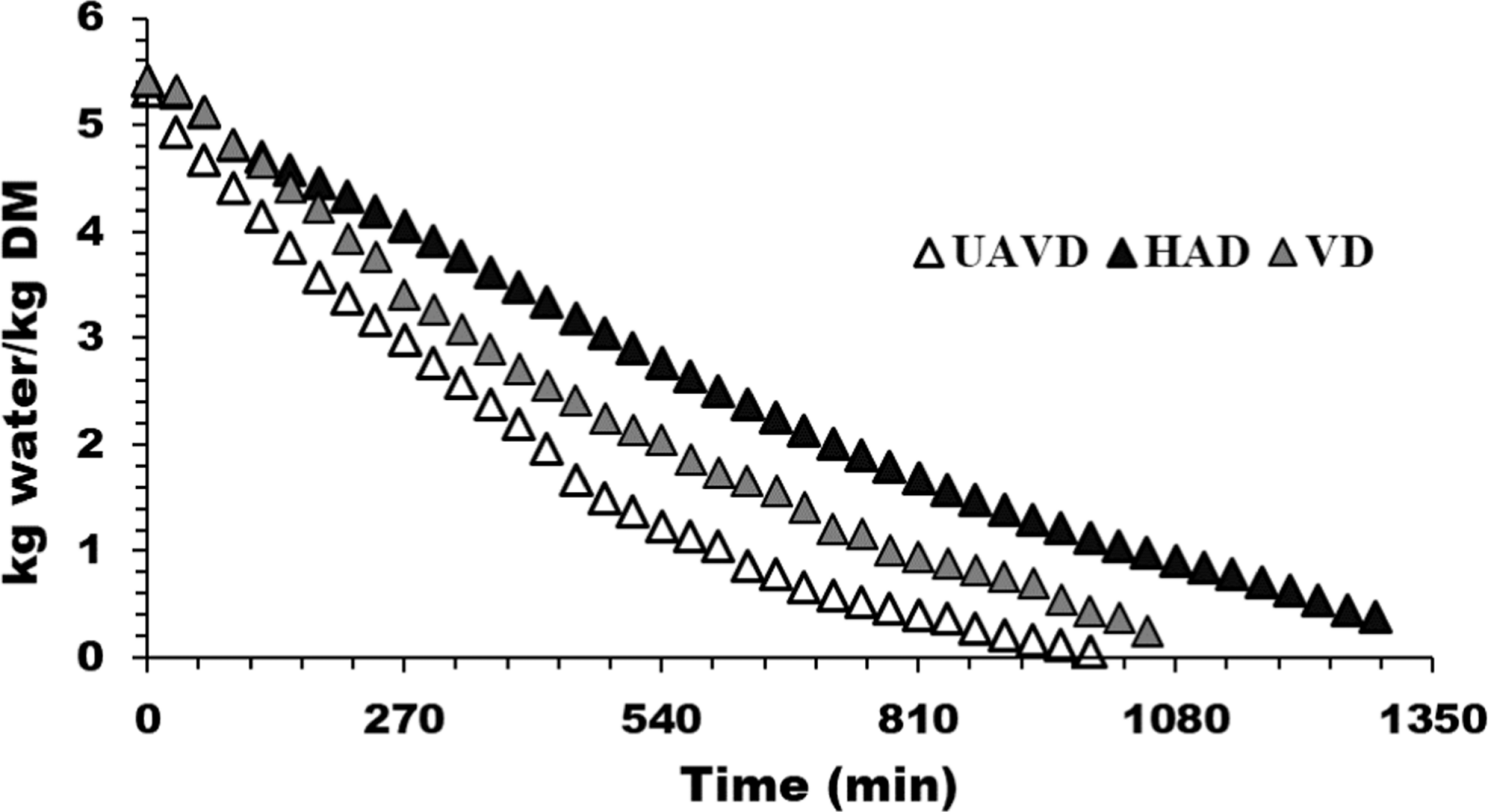
Drying curves of blueberries. HAD: hot air drying; VD, vacuum drying; UAVD, ultrasound-assisted vacuum drying.

The initial moisture content of blueberries was determined to be 83.95 ± 1.15%. The drying curves of three drying methods HAD, VD, and UAVD are presented in Figure 1. The drying process continued until the moisture content reached 0.2 kg water/kg DM. Also, the drying times were 1290, 1050, and 990 min for HAD, VD, and UAVD at 50 °C, respectively. In the drying period of both methods, the constant rate period was shorter compared to the falling rate period, indicating that a diffusion mechanism primarily governed the moisture transfer,^36^ especially in HAD. UAVD displayed the shortest drying period compared to VD and HAD. This result could be explained by the fact that HAD took a longer time to achieve dynamic equilibrium due to the internal moisture transport within the sample, making it a time-consuming procedure and leading to a gradual moisture migration from the interior.^37,38^ The drying time of UAVD was approximately 1.30 times less than that of HAD and 1.06 times less than that of VD. The utilization of ultrasound enables efficient moisture removal by facilitating rapid internal moisture transfer through a series of compressions and expansions within the medium. Additionally, the creation of cavitation aids in the effective extraction of tightly bound moisture without substantial heating, preserving the integrity of heat-sensitive food components.^39^ The resulting explosive force creates microchannels, reducing moisture diffusion resistance and consequently decreasing drying time.^40^ Various studies have demonstrated that ultrasound-assisted drying increases the drying rate for a wide range of products, including red peppers, green beans, nectarine, hawthorn juices, and melon.^17,19,36,39,41^

#### 3.1.1 Modeling of Drying Behavior

Table 1 presents eight thin-layer drying models utilized for simulating blueberry drying behavior. Model parameters, along with *R*^2^ and RMSE values, are listed in Table 2. To identify the most suitable mathematical model for fitting experimental data, both the maximized *R*^2^ and the minimized RMSE were considered. As a result, the two-term and Weibull models were selected as the best models to represent the drying behavior of blueberries. The *R*^2^ values of the models ranged from 0.853 to 0.999, indicating the successful application of drying models to describe the drying behavior of the blueberry samples.

**Table 2:** Drying model coefficients for selected models

		model parametres					
model	*R*^2^	*k* × 1000	RMSE	a	b	c	d
HAD							
Lewis	0.856	172.45	0.132				
Henderson and Pabis	0.917	100.26	0.070	0.644			
logarithmic	0.917	83.00	0.070	0.690		–0.059	
two term	0.984		0.031	0.560	0.440	0.085	31.797
Wang and Singh	0.953		0.156	–0.124	0.004		
two-term exp.	0.981	779.88	0.110	0.183			
parabolic	0.914		0.071	0.602	0.602	0.001	
Weibull	0.948		0.056	0.414	3.918		
VD							
Lewis	0.989	114.45	0.043				
Henderson and Pabis	0.994	124.67	0.032	1.080			
logarithmic	0.999	78.35	0.010	1.306		–0.275	
two term	0.994		0.032	0.625	0.456	0.125	0.125
Wang and Singh	0.999		0.013	–0.087	0.002		
two-term exp	0.999	166.36	0.016	1.821			
parabolic	0.999		0.011	1.017	–0.091	0.002	
Weibull	0.999		0.014	1.273	8.771		
UAVD							
Lewis	0.938	83.83	0.105				
Henderson and Pabis	0.959	100.40	0.085	1.155			
logarithmic	0.995	3.60	0.030	17.680		–16.610	
two term	0.959		0.085	0.590	0.564	0.100	0.100
Wang and Singh	0.996		0.029	–0.042	–0.001		
two-term exp	0.989	154.58	0.046	2.149			
parabolic	0.997		0.025	1.038	–0.051	–0.001	
Weibull	0.997		0.023	1.949	10.918		

Different lowercase letters in the same line indicate differences between samples subjected to different drying methods (*p* < 0.05). HAD, hot air drying; UAVD, ultrasound-assisted vacuum drying, VD, vacuum drying.

### 3.2 Effects of Drying Methods on Bioactive Compounds

The total bioactive content of blueberries is influenced by various factors, including the species and cultivar, maturity, climate, plant location, and cultivation year.^42,43^ The total phenolic content (TPC) and antioxidant capacity of fresh and dried blueberries are given in Table 3. The TPC value of fresh blueberries was found to be 1423.31 mg GAE/100 g DM, while the TPC values of dried samples were found to be 347.38–1662.83 mg GAE/100 g DM. The drying methods significantly affected the TPC value (*p* < 0.05). The TPC values of the dried samples with the exception of FD dried ones were lower than those of fresh samples, indicating that the degradation in phenolic compounds occurred especially in HAD methods.

**Table 3:** Total Phenolic Content and Antioxidant Activity Values of Fresh and Dried Blueberry

			dried blueberry		
bioactive properties	fresh blueberry	HAD	VD	UAVD	FD
TPC (mg GAE/100 g DM)	1423.31 ± 38.62^b^	347.38 ± 7.65^e^	564.88 ± 19.13^d^	809.77 ± 7.65^c^	1662.83 ± 18108^a^
DPPH (mg TE/100 g DM)	131.42 ± 1.23^b^	57.46 ± 0.24^d^	57.63 ± 0.95^d^	60.64 ± 0.47^c^	171.25 ± 1.18^a^
ABTS (mg TE/100 g DM)	2.41 ± 0.01^d^	2.34 ± 0.01^e^	2.59 ± 0.00^c^	3.03 ± 0.08^b^	4.09 ± 0.01^a^

Different lowercase letters in the same line indicate differences between samples subjected to different drying methods (*p* < 0.05). FD, freeze-drying; HAD, hot air drying; VD, vacuum drying; TPC, total phenolic content; UAVD, ultrasound-assisted vacuum drying.

In FD, the drying process occurs at a very low temperature and under vacuum conditions;^44^ therefore, phenolic compounds may be protected. The use of UAVD provides higher retention of TPC compared to HAD and VD. The greater TPC value of the samples dried with UAVD might be explained by the fact that ultrasound cavitation can boost the extraction rate of phenolic compounds by breaking down plant cells, which improves solvent penetration.^45^ Many studies reported that ultrasound-assisted vacuum drying causes lower degradation in Asian pear,^46^ red peppers,^17^ nectarines.^19^ The samples dried with HAD showed the lowest TPC value compared to other samples. Phenolic compounds are susceptible to thermal and oxidative reactions. The longer drying time, temperature, and oxidative reaction during HAD treatment may have caused a significant reduction in phenolic compounds.

The antioxidant capacity of blueberries was determined using DPPH and ABTS methods, showing values ranging from 57.46 to 171.25 mg of TE/100 g of DM and 2.34 to 4.09 mg of TE/100 g of DM, respectively. Like the TPC results, FD blueberries exhibited the highest antioxidant capacity for DPPH and ABTS. Also, the results showed that significant differences were observed between fresh and dried blueberries (*p* > 0.05). UAVD samples showed higher antioxidant activity compared to VD and HAD. Similar results for UAVD drying raspberries^21^ and Asian pear^46^ were reported. The study also found strong correlations and relationships between TPC and ABTS (0.65), TPC and DPPH (0.96), and DPPH and ABTS (0.66). Similar results were reported by ref (39) for TPC and DPPH. A balance is maintained in the levels of compounds and antioxidant activity values by the interaction of mechanisms that increase concentration (e.g., water evaporation leading to a concentration impact) and those that decrease it (such as the degradation reaction of phenolic compounds).^39^ In conclusion, the study suggests that UAVD can be considered an alternative method for drying blueberries due to its ability to retain phenolic compounds while subjecting the fruit to less heat treatment.

### 3.3 Anthocyanin Profiles

Table 4 displays the anthocyanin profiles of samples of dried and fresh blueberries. The blueberries are rich in anthocyanins such as cyanidins, delphinidins, malvidins, petunidins, and peonidins.^47^ The major anthocyanin identified for blueberry was malvidin-3-*O*-galactoside and delphinidin-3-*O*-galactoside, which is consistent with previous studies.^48,49^ The recorded values for cyanidin-3-glucoside per 100 g of fresh weight (FW) range from 19.3 to 677.8 mg, cyanidin-3,5-glucoside from 456.7 to 1406.3 mg, cyanidin-3-glucoside from 44.3 to 417.7 mg, and malvidin-3-glucoside from 101.88 to 195.01 mg.^50,51^ Variations in the total anthocyanin content among cultivars are primarily attributed to differences in genotypes and the environmental growing conditions.^50^

**Table 4:** Individual Anthocyanin of Fresh and Dried Blueberry Samples

	drying methods				
anthocyanins	fresh	HAD	VD	UAVD	FD
delphinidin-3-*O*-galactoside	5.61 ± 0.02^c^	1.08 ± 0.06^d^	5.17 ± 0.06^c^	15.29 ± 0.76^b^	49.8 ± 0.16^a^
delphinidin-3-*O*-glucoside	0	0	0	0.36 ± 0.03^b^	0.8 ± 0.02^a^
cyanidin-3-*O*-galactoside	21.19 ± 0.84^b^	2.76 ± 0.05^e^	8.30 ± 0.05^d^	13.96 ± 0.18^c^	49.3 ± 0.12^a^
delphinidin-3-*O*-arabinoside	3.73 ± 0.04^c^	0.48 ± 0.02^e^	3.25 ± 0.02^d^	7.70 ± 0.03^b^	28.9 ± 0.64^a^
cyanidin-3-*O*-glucoside	0.62 ± 0.01^b^	0.12 ± 0.01^d^	0.48 ± 0.01^c^	0.60 ± 0.01^b^	1.7 ± 0.04^a^
petunidin-3-*O*-galactoside	8.72 ± 0.05^c^	1.2 ± 0.06^e^	5.53 ± 0.14^d^	11.92 ± 0.02^b^	34.0 ± 0.08^a^
cyanidin-3-*O*-arabinoside	14.33 ± 0.23^b^	1.08 ± 0.05^e^	4.81 ± 0.06^d^	7.10 ± 0.08^c^	26.1 ± 0.07^a^
paeonidin-3-*O*-galactoside	12.46 ± 0.32^b^	1.32 ± 0.01^e^	4.69 ± 0.05^d^	7.10 ± 0.02^c^	19.8 ± 0.18^a^
petunidin-3-*O*-arabinoside	5.61 ± 0.04^b^	0.48 ± 0.03^d^	2.52 ± 0.01^c^	5.17 ± 0.02^b^	16.0 ± 0.15^a^
malvidin-3-*O*-galactoside	66.68 ± 0.48^b^	5.29 ± 0.03^e^	25.76 ± 0.03^d^	45.39 ± 0.05^c^	102.3 ± 0.21^a^
malvidin-3-*O*-glucoside	8.72 ± 0.52^b^	0.60 ± 0.02^e^	2.88 ± 0.03^d^	4.33 ± 0.06^c^	11.7 ± 0.49^a^
malvidin-3-*O*-arabinoside	42.38 ± 0.27^b^	2.52 ± 0.05^e^	13.36 ± 0.05^d^	21.07 ± 0.05^c^	54.3 ± 0.14^a^

Different lowercase letters in the same line indicate differences between samples subjected to different drying methods (*p* < 0.05). FD, freeze-drying; HAD, hot air drying; VD, vacuum drying; UAVD, ultrasound-assisted vacuum drying. C3GE, cyanidin-3-glucoside equivalent.

The malvidin-3-*O*-galactoside content ranged from 5.29 to 102.3 mg/100 g, indicating a high level of anthocyanin in blueberry. The anthocyanin content in dried samples was lower than that in the fresh sample except for the FD dried sample. Anthocyanins have low thermal stability, leading to the thermal degradation of anthocyanins during HAD, VD, and UAVD.^52^ The higher degradation occurred in samples dried with HAD. This could be due to the long drying time and high thermal load, resulting in significant anthocyanin losses in the HAD and VD, and technique.^21^ FD exhibited the highest anthocyanin content, followed by UAVD. The FD process occurred under a vacuum at low temperature and could not lead to thermal and oxidative degradation in anthocyanins.^53^ The UAVD technique allows us to increase the extraction efficiency and disruption of cell walls by cavitation effects, which means improved mass transfer and increased interaction surface area between the solvent and anthocyanins.^54^ These results highlight the potential of UAVD, FD, and VD as important alternatives for preserving anthocyanins in blueberry samples.

### 3.4 Color Characteristics

**Figure 2:**
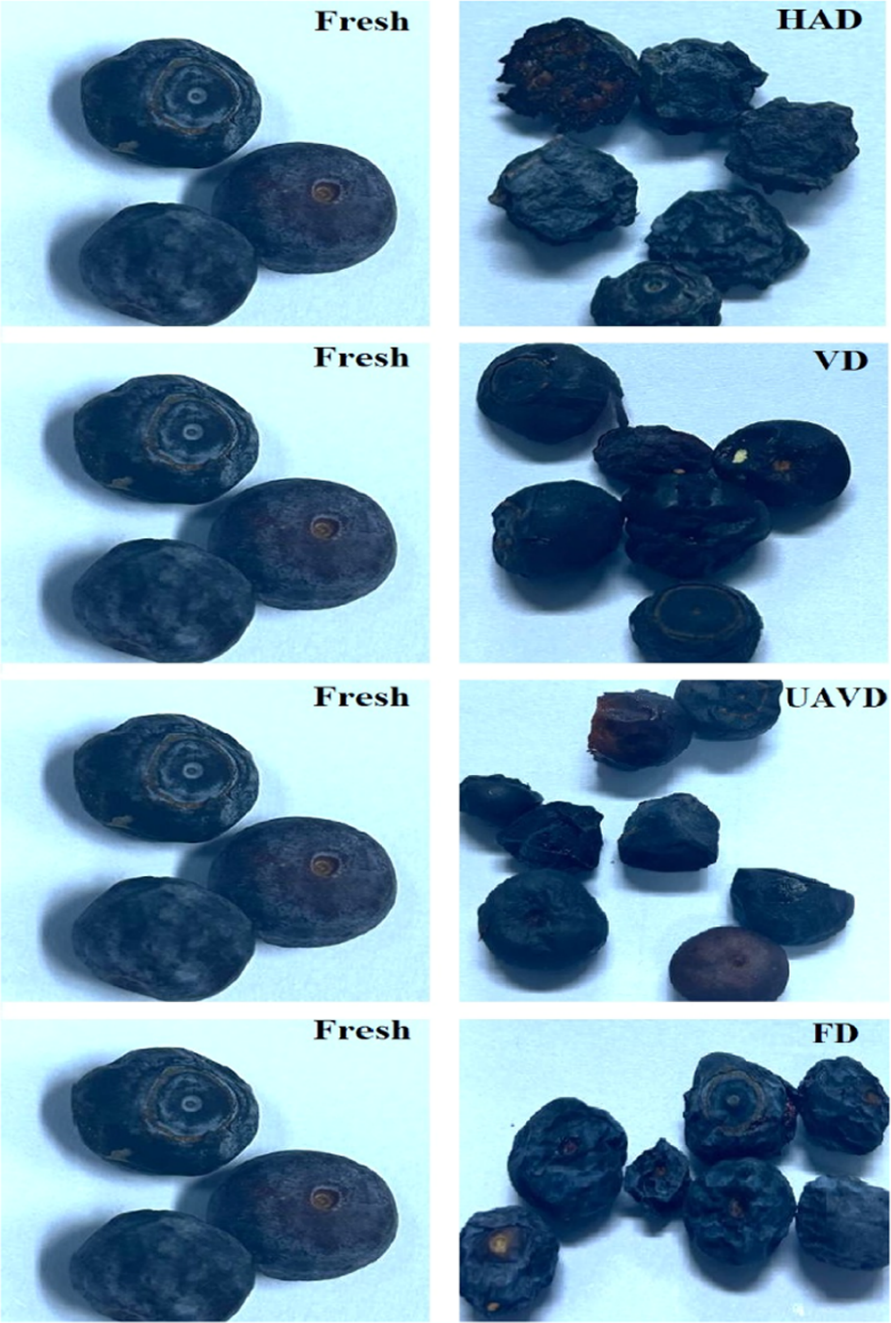
Fresh and dried blueberry.

The images of the dried samples are given in Figure 2. Table 5 presents the effects of various drying methods on the color parameters of the blueberries. The initial L*, a*, and b* values for fresh blueberry samples were measured as 35.31, 11.38, and 3.51, respectively. However, UAVD blueberries displayed a significant decrease in L*, a*, and b* values. HAD and VD blueberries showed nonsignificant differences in L* and b* values. The *L* value of the samples treated with FD was found to be significantly higher than that of the fresh sample (*p* < 0.05), while the *L* value of the samples treated with UAVD was found to be significantly lower than that of the fresh sample (*p* < 0.05). There was no significant difference between the *L* values of the samples dried with VD and HAD and that of the fresh sample. The effect of drying methods on the L* value can be explained through different mechanisms. Nonenzymatic browning reactions do not occur in samples treated with FD.^55^ With the reflection of light on the surface with drying, a brighter appearance may have occurred in the FD-treated samples. In addition, the increase in the concentration of the white waxy layer on the outer part of the blueberry fruit with drying may have caused an increase in the L* value in the samples dried with FD.^56^ The removal of the white waxy layer and the occurrence of nonenzymatic browning reactions caused a decrease in the L* value in the samples dried with UAVD. This result is clearly observed in the photographs of the dried samples. The concentration of the waxy layer in the samples treated with VD and HAD increased. However, the L* value of the samples dried with VD and HAD did not change significantly (*p* < 0.05). The nonenzymatic reaction during HAD and VD caused a limited increase in the L* value of the dried fruits.^57^

**Table 5:** Color Parameters of Fresh and Dried Blueberries

			dried blueberry		
color parameters	fresh blueberry	HAD	VD	UAVD	FD
L*	35.31 ± 1.49^b^	35.74 ± 0.90^b^	38.95 ± 0.29^b^	31.78 ± 0.31c	43.63 ± 1.24^a^
a*	11.38 ± 1.01^b^	8.6 ± 0.45^d^	12.31 ± 0.22^b^	10.13 ± 0.18^c^	16.25 ± 0.65^a^
b*	3.51 ± 0.84^b^	2.37 ± 0.63^bc^	5.69 ± 0.51^b^	3.41 ± 0.01^c^	8.62 ± 2.04^a^
Δ*E*		2.58	3.68	3.02	8.11

Different lowercase letters in the same line indicate differences between samples subjected to different drying methods (*p* < 0.05). FD, freeze-drying; HAD, hot air drying; VD, vacuum drying; UAVD, ultrasound-assisted vacuum drying.

The a* values of the FD were found to be higher than those of the fresh samples due to the reduced degradation of the anthocyanin.^58^ The lowest Δ*E* value was found for UAVD (3.03), while the highest value was observed for FD (10.92). This difference in Δ*E* is attributed to the concentration of pigments present in FD. Δ*E* values greater than 5 indicated a noticeable color change after drying.^59^ The drying methods led to changes in the cell structure of the sample and partially harmed and modified. The color differences of Δ*E* could be explained by this modification.^60,61^

### 3.5 Microstructure of Dried Blueberries

**Figure 3:**
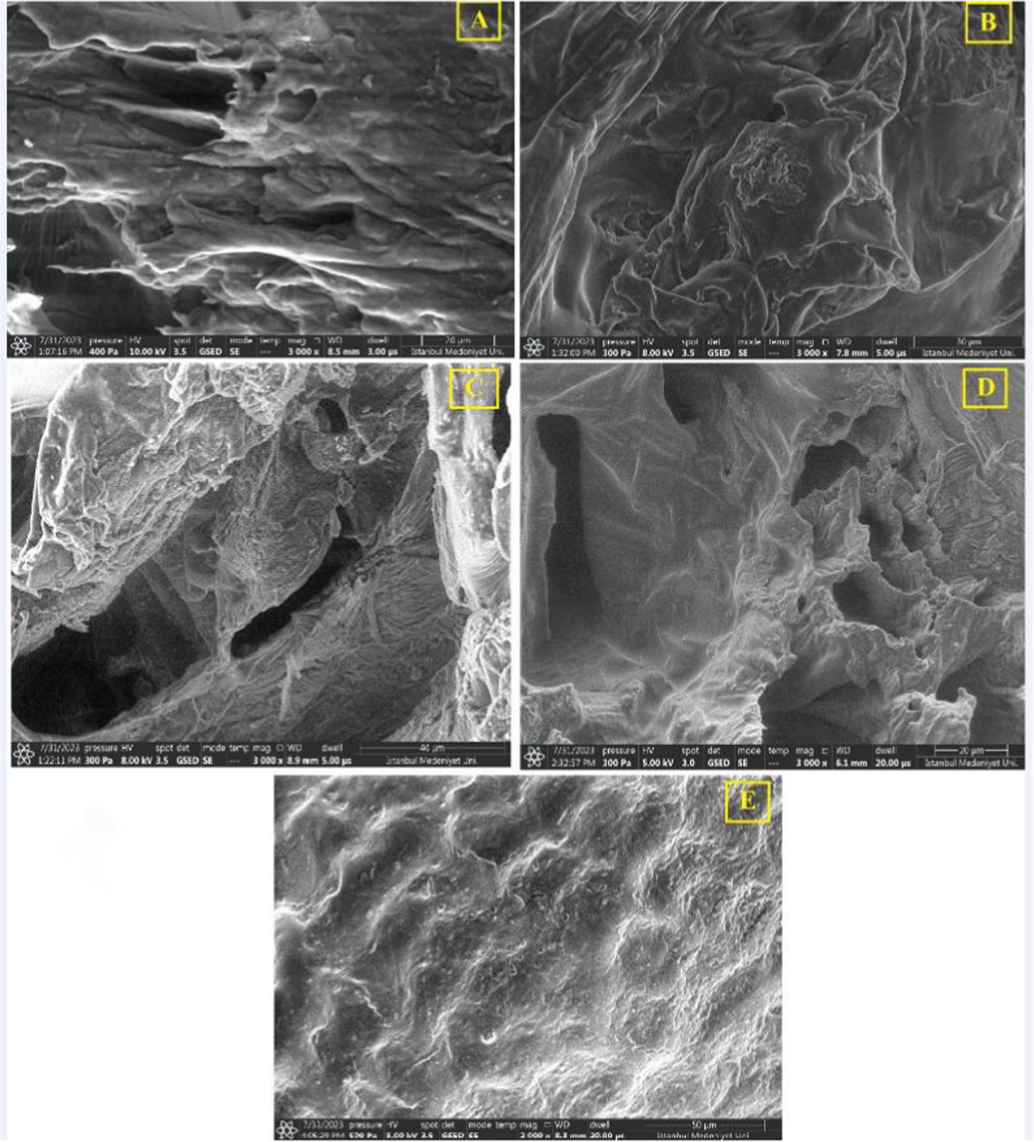
SEM images of dried blueberries subjected to (A) freeze-drying, (B) hot air drying, (C) ultrasound-assisted vacuum drying, and (D) vacuum drying and (E) fresh blueberries.

The SEM images of the dried blueberries are given in Figure 3. As seen, the blueberry samples were partially damaged by HAD and underwent tissue breakage.^21^ The blueberry samples with UAVD showed more porosity and open structure compared to those with VD because of lower drying time and cavitation effects led to evaporation of water on the inner surface.^62^ The blueberry with FD showed more porosity and higher open structure than those with VD and UAVD. This result could be explained by the fact that the porosity and open structure were less harmed at low temperatures.^62^ In addition, UAVD and FD showed less shrinkage and cell damage. Similar results were reported by ref (21) for the raspberry with UAVD and FD.

## 4 Conclusions

In this study, the influence of hot air drying, ultrasound-assisted vacuum drying, and freeze-drying methods on the drying characteristics, bioactive compounds, anthocyanin content, microstructural properties, and color properties of blueberries were examined. In comparison to VD (1050 min) and HAD (1290 min), ultrasound-assisted vacuum drying (UAVD, 990 min) significantly reduced the drying time. Due to their higher *R*^2^ values compared to other drying models, Weibull and two-term exponential drying models can be chosen as the best drying models to represent the drying behavior of blueberry samples. In comparison to HAD and VD, the results indicated that the application of UAVD increased the retention of TPC. Blueberries with FD had the best antioxidant capacity for DPPH and ABTS. This study suggested that UAVD could be used as an alternative method to HAD due to its low drying time and higher bioactive retention than HAD.
